# Demonstrating the Protective Efficacy of the Novel Fluoroquinolone Finafloxacin against an Inhalational Exposure to Burkholderia pseudomallei

**DOI:** 10.1128/AAC.00082-17

**Published:** 2017-06-27

**Authors:** Kay B. Barnes, Karleigh A. Hamblin, Mark I. Richards, Thomas R. Laws, Andreas Vente, Helen S. Atkins, Sarah V. Harding

**Affiliations:** a CBR Division, Defence Science and Technology Laboratory, Porton Down, Salisbury, United Kingdom; b MerLion Pharmaceuticals, Berlin, Germany; c University of Exeter, Exeter, United Kingdom

**Keywords:** Burkholderia, finafloxacin, aerosol, mouse model

## Abstract

Burkholderia pseudomallei is the causative agent of melioidosis, a serious disease endemic in Southeast Asia and Northern Australia. Antibiotic treatment is lengthy and relapse often occurs. Finafloxacin is a novel fluoroquinolone with increased antibacterial activity in acidic conditions in contrast to other fluoroquinolones which demonstrate reduced activity at a lower pH. Therefore, finafloxacin may have improved efficacy against B. pseudomallei, which can survive within host cells where the local pH is acidic. *In vitro* analysis was performed using MICs, minimal bactericidal concentrations (MBCs), time-kill assays, persister cell assays, and macrophage assays. Finafloxacin showed increased bactericidal activity at pH 5 in comparison to pH 7 and ciprofloxacin at pH 5. *In vivo* studies in BALB/c mice included pharmacokinetic studies to inform an appropriate dosing regimen. Finafloxacin efficacy was evaluated in an inhalational murine model of melioidosis where antibiotic treatment was initiated at 6 or 24 h postchallenge and continued for 14 days, and mice were observed for 63 days. The survival of infected mice following 14 days of treatment was 80%, 60% or 0% for treatments initiated at 6 h and 60%, 30% or 0% for treatments initiated at 24 h for finafloxacin, co-trimoxazole, or ciprofloxacin, respectively. In summary, finafloxacin has increased bactericidal activity for B. pseudomallei under acidic conditions *in vitro* and improves survival in a murine model of melioidosis compared with those for ciprofloxacin. Furthermore, finafloxacin improves bacteriological clearance compared with that of co-trimoxazole, suggesting it may offer an effective postexposure prophylaxis against B. pseudomallei.

## INTRODUCTION

Burkholderia pseudomallei is a Gram-negative environmental saprophyte found in soil and water in tropical and subtropical areas of the world (1). It is the causative agent of melioidosis, a serious disease that affects humans and many other animal species (2). Melioidosis is endemic in Southeast Asia and Northern Australia; however, new foci of infection are regularly being identified (2, 3). The main routes of infection with B. pseudomallei are cutaneous inoculation, inhalation, aspiration, and oral inoculation; the most common route is thought to be by cutaneous inoculation, mainly due to the primary reservoir being standing water, such as rice paddy fields and moist tropical soil (2, 4). In addition, the U.S. Centers for Disease Control and Prevention classify B. pseudomallei as a category B biological agent, and it is considered a candidate for deliberate release, most likely in the form of an aerosol (5). Inhalation of B. pseudomallei usually leads to more severe disease; therefore, early treatment is paramount and an effective antibiotic prophylaxis is required (6).

B. pseudomallei is an intracellular pathogen. Invasion of both phagocytic and nonphagocytic cells occurs, and following internalization, B. pseudomallei can be found within phagosomes and phagolysosomes, where it is able to survive despite these being acidified environments with a pH as low as 5.0 (7–10). The organism can escape from the phagolysosome and enter the cytoplasm, where it is able to replicate. In B. pseudomallei-infected RAW 264.7 macrophages, 71% and 29% of bacteria were shown to be in the cytoplasm and within phagosomes, respectively, 6 h following infection (11).

Clinical manifestations of melioidosis are diverse, ranging from asymptomatic latent infection or chronic manifestations with the formation of multiple abscesses to the presentation of acute pneumonia or sepsis, which often results in death within a few days of exposure (12, 13). Mortality rates for patients with melioidosis remain high at 10 to 20% in Australia and 40% in Thailand, despite antibiotic intervention (12). A recent study detailed that recurrent melioidosis occurred in 10% of cases when patients were followed over a 3-year period, although 52% of these were shown to be due to reinfection (14). Demonstrating the potential for long-term relapse, an incubation period of 62 years has been recorded, where reactivation of disease was due to a separate traumatic incident (15). It is also believed that melioidosis is underreported in many countries, particularly those with poor diagnostic capabilities (16).

Melioidosis is intrinsically resistant to many antimicrobial agents, particularly in anaerobic acidic conditions that may be encountered by B. pseudomallei in vivo (14). The current recommended treatments are derived from a series of large randomized clinical trials performed in Northeast Thailand (14, 17). Treatment is divided into two phases, namely, the acute phase, aimed at preventing death from overwhelming sepsis, and the oral eradication phase, aimed at killing residual bacteria and minimizing the risk of relapse. The acute phase typically involves the administration of parenteral ceftazidime or meropenem for 10 to 14 days (14). Many antibiotics have been used in the oral eradication phase, including co-trimoxazole, chloramphenicol, co-amoxiclav, and doxycycline administered alone or in various combinations (13, 14). However, the risk associated with side effects is high with these combinations, and the results from the recent MERTH randomized clinical study recommends co-trimoxazole alone to be delivered orally for 12 to 20 weeks (17).

The existing fluoroquinolones ciprofloxacin and ofloxacin have been used to treat melioidosis patients as a component of the eradication-phase treatment. In an open pilot study of 57 patients, there were 13 treatment failures, of which 5 patients failed to respond to treatment and a further 8 patients relapsed, leading to an overall failure rate of 29% (18). For these reasons, regimens containing fluoroquinolones are not currently recommended for treating melioidosis unless there is resistance to or intolerance of other available antibiotics (14, 18). In the event of a deliberate release or accidental exposure in a laboratory, postexposure prophylaxis (PEP) is relied upon (19). However, there is currently no available data on the evaluation of antibiotics for PEP in humans. On the basis of data generated from animal studies, the United Kingdom recommends co-trimoxazole (960 mg delivered orally twice daily) for 7 days (20). Other international recommendations state that co-trimoxazole should be given for 21 days (960-mg tablets, 2 every 12 h) (14).

**FIG 1:**
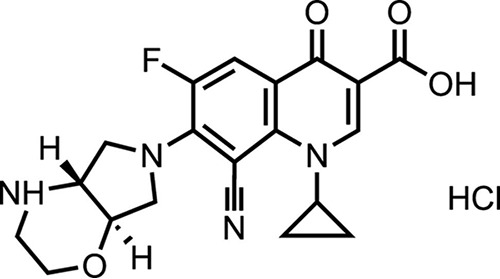
Chemical structure of finafloxacin.

There is an ongoing requirement for novel antibiotic formulations for effective PEP and treatment of B. pseudomallei infection. Finafloxacin is a novel C-8-cyano-fluoroquinolone containing a unique chiral C-7 substituent (Fig. 1); this unique property confers enhanced activity under acidic conditions where other fluoroquinolones, including ciprofloxacin, are inactivated (21, 22). Therefore, finafloxacin may exhibit advantages over other fluoroquinolones against bacteria that reside within acidic cellular organelles, such as phagosomes and phagolysosomes (23). Finafloxacin is currently under development by MerLion Pharmaceuticals Pte Ltd. for the treatment of serious infections with acidic foci (21, 24, 25). A finafloxacin otic suspension received its first global approval by the United States Food and Drug Administration in December 2014 (26). A phase II clinical study with oral and intravenous formulations of finafloxacin for the treatment of complicated urinary tract infections and pyelonephritis has been completed successfully (24, 27). Several preclinical and clinical studies have demonstrated that finafloxacin has increased bactericidal activity at an acidic pH against ciprofloxacin-resistant strains of Escherichia coli and Acinetobacter baumannii, intracellular Legionella pneumophila, and other Gram-negative and Gram-positive bacterial species (21–23, 28–30).

The mechanism of action of the fluoroquinolone class of antibiotics is to inhibit DNA synthesis by promoting cleavage of bacterial DNA in the DNA-enzyme complexes of DNA gyrase and topoisomerase IV, leading to rapid bacterial death (31, 32). The concentration of finafloxacin required to achieve 50% DNA cleavage in the presence of DNA gyrase or topoisomerase IV was 12-fold or 25-fold less than that for ciprofloxacin, respectively, demonstrating an increased potency (33). Thus, in addition to having potent bactericidal activity under acidic conditions, finafloxacin also shows increased potency against DNA gyrase and topoisomerase IV, suggesting that finafloxacin may offer improved efficacy against B. pseudomallei where other fluoroquinolones have limited effects (34).

Despite the fluoroquinolone ciprofloxacin demonstrating good *in vitro* activity against B. pseudomallei, it is known that *in vivo* efficacy is incomplete, which may be attributed to ciprofloxacin having reduced antibacterial activity in acidic conditions (18, 34). Since finafloxacin has increased bactericidal activity against other Gram-negative bacteria at acidic pHs and may offer improved efficacy against B. pseudomallei (28), an aim of this work was to assess the *in vitro* activity of finafloxacin in both neutral and acidic conditions in comparison to that of ciprofloxacin. Furthermore, the unique property of finafloxacin having activity at an acidic pH may enable the elimination of bacteria from the site of infection and, if used as PEP, may prevent the establishment of a persistent infection. To investigate the *in vivo* efficacy for B. pseudomallei infection, PEP with finafloxacin was evaluated in a murine model of inhalational melioidosis in comparison to that with ciprofloxacin and co-trimoxazole.

## RESULTS

### MICs and MBCs.

MICs and minimal bactericidal concentrations (MBCs) were used to quantify the concentrations of antibiotic required to inhibit or kill bacteria, respectively. In all assays, sodium hydroxide at the same concentration used to dissolve the antibiotics was included as a control. MIC assays were performed in Mueller-Hinton broth (MHB) adjusted to pH 5 or pH 7 for finafloxacin and ciprofloxacin against B. pseudomallei strains K96243 and 576. Finafloxacin demonstrates activity against B. pseudomallei at pH 7 with an MIC of 4 μg/ml; however, at pH 5, it has a lower MIC of 1 to 2 μg/ml (Table 1). Conversely, ciprofloxacin has a higher MIC at pH 5 (32 μg/ml) than at pH 7 (1 to 2 μg/ml). The MIC of finafloxacin was lower for both strains of B. pseudomallei at pH 5 in comparison to that at pH 7, demonstrating improved antibacterial activity at a lower pH. By contrast, the activity of ciprofloxacin was greatly reduced at pH 5 with a 32-fold increase in the MIC observed for both strains of B. pseudomallei, suggesting that the antibacterial activity of ciprofloxacin is reduced under these acidic conditions. Similar to the MIC assays, in MBC assays, finafloxacin demonstrated greater bactericidal activity at pH 5 (4 to 8 μg/ml) than at pH 7 (16 μg/ml), and ciprofloxacin demonstrated lower bactericidal activity at pH 5 (32 μg/ml for strain K96243 and >64 μg/ml for strain 576) than at pH 7 (8 to 16 μg/ml) (Table 1).

**TABLE 1:** MICs and MBCs of finafloxacin and ciprofloxacin for B. pseudomallei^*a*^

Organism	MIC (μg/ml)				MBC (μg/ml)			
	Finafloxacin		Ciprofloxacin		Finafloxacin		Ciprofloxacin	
	pH 5	pH 7	pH 5	pH 7	pH 5	pH 7	pH 5	pH 7
B. pseudomallei K96243	1	4	32	1	8	16	32	8
B. pseudomallei 576	2	4	32	1	4	16	>64	16

a MIC assays were performed in MHB adjusted to pH 5 or pH 7. MBCs were determined by plating on L agar.

### Time-kill assays.

Time-kill assays were used to measure the activity of finafloxacin over time. Here, finafloxacin or ciprofloxacin at 4× MIC in medium at pH 7 (16 μg/ml or 4 μg/ml, respectively) (Table 1) was added to L broth adjusted to pH 7, and the medium was inoculated with B. pseudomallei strains K96243 or 576 and sampled over a 24-h period. To compare the levels of activity under acidic conditions, the time-kill assay was repeated using the same antibiotic concentrations (finafloxacin at 16 μg/ml or ciprofloxacin at 4 μg/ml) with the medium adjusted to pH 5.

**FIG 2:**
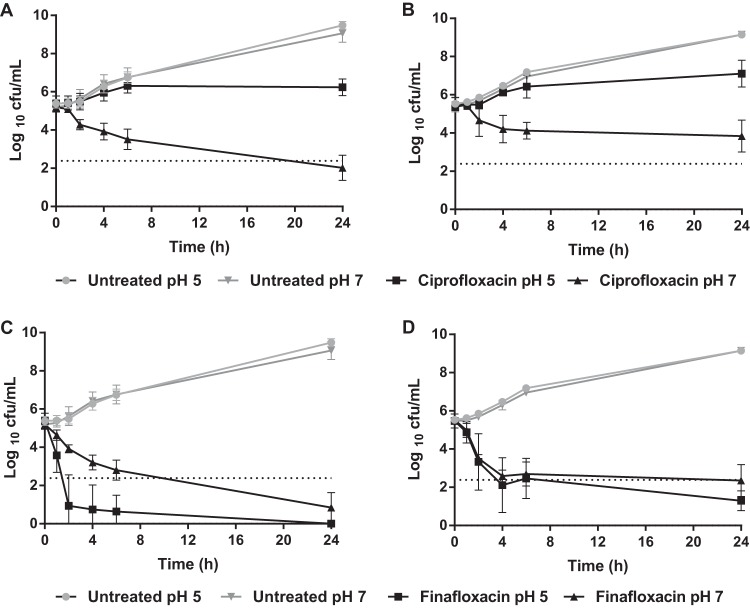
Effect of pH on the efficacy of ciprofloxacin and finafloxacin against B. pseudomallei strains over a 24-h period. Time-kill assays were performed in L broth adjusted to pH 5 or pH 7 with finafloxacin (16 μg/ml) or ciprofloxacin (4 μg/ml). An untreated bacterial control was included. (A) Ciprofloxacin against B. pseudomallei strain K96243. (B) Ciprofloxacin against B. pseudomallei strain 576. (C) Finafloxacin against B. pseudomallei strain K96243. (D) Finafloxacin against B. pseudomallei strain 576. The error bars represent the standard errors of the mean (SEMs) from 3 biological replicates. ┈, 3-log_10_ reduction in CFU/ml from the starting inoculum.

At pH 7, ciprofloxacin demonstrated bactericidal activity over 24 h for strain K96243 but showed bacteriostatic activity against strain 576 (Fig. 2A and B). However, at pH 5, ciprofloxacin showed bacteriostatic activity against both strains of B. pseudomallei. As for the MICs, ciprofloxacin was significantly more active in medium adjusted to pH 7 than to pH 5 (*P* < 0.0001). By comparison, finafloxacin demonstrated bactericidal activity over 24 h for both strains of B. pseudomallei at both pHs, with a greater than 3-log_10_ reduction in CFU/ml compared with the starting inoculum (Fig. 2C and D). At 24 h, finafloxacin was more active at pH 5 than at pH 7 for strain 576 (*P* < 0.01). Impressively, finafloxacin demonstrated a rapid bactericidal activity against strain K96243 and was significantly more active at pH 5 than at pH 7 at all time points in the assay (*P* < 0.0001), with no bacteria recoverable at 24 h. These time-kill data consolidate the finding that finafloxacin, unlike ciprofloxacin, demonstrates greater bactericidal activity under acidic conditions than under neutral conditions.

### Macrophage assays.

**FIG 3:**
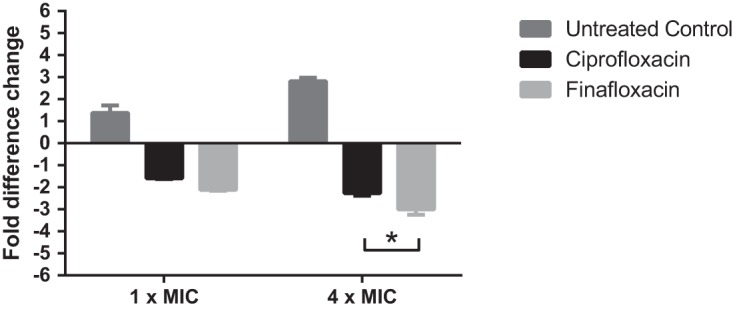
Fold difference change in finafloxacin- and ciprofloxacin-treated J774 macrophages infected with B. pseudomallei strain AI. Macrophages were infected and incubated with L 15 medium at 37°C containing finafloxacin (16 μg/ml or 4 μg/ml for 4× MIC or 1× MIC, respectively) or ciprofloxacin (4 μg/ml or 1 μg/ml for 4× MIC or 1× MIC, respectively) over a 24-h period. An untreated bacterial control was also included. The error bars represent the SEMs from 3 biological replicates. Statistical analysis was performed by 2-way ANOVA with experimental run used as a repeated measure, and fold change in bacterial numbers is graphed. *, *P* < 0.05.

Macrophage infection assays were performed to determine the intracellular activity of finafloxacin for B. pseudomallei in comparison to that of ciprofloxacin. The assays required the bacteria in the extracellular supernatant to be killed using gentamicin; as B. pseudomallei is naturally resistant to gentamicin, a gentamicin-sensitive B. pseudomallei efflux pump mutant strain AI (deficient in the *amrA* gene) was used. At 1× and 4× MICs, both finafloxacin and ciprofloxacin significantly reduced the intracellular bacterial concentrations in comparison to those of untreated cells (*P* < 0.0001) (Fig. 3). At 1× MIC, there was no significant difference between the activities of ciprofloxacin and finafloxacin (*P* > 0.05). However, at 4× MIC, finafloxacin significantly reduced the intracellular bacterial concentration over 24 h compared with that by ciprofloxacin (*P* < 0.05).

### Persister cell assays.

**FIG 4:**
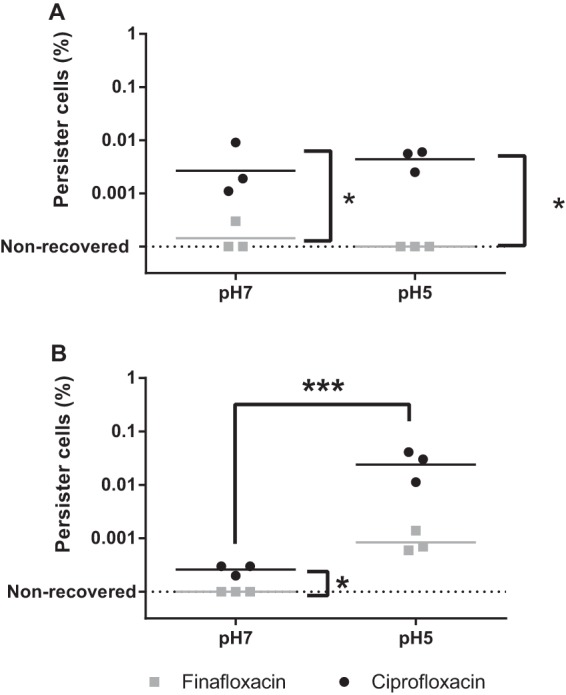
Effects of finafloxacin and ciprofloxacin on the formation of B. pseudomallei persister cells. The percentages of B. pseudomallei persister cells produced when strain 576 (A) or strain K96243 (B) was incubated with 100× MIC of finafloxacin (400 μg/ml) or ciprofloxacin (100 μg/ml) for 24 h. These data were analyzed using repeated measures (accounting for experimental run) ANOVA. *, *P* < 0.05; ***, *P* < 0.001.

It is hypothesized that B. pseudomallei has the ability to form persister cells *in vivo*, contributing to the difficulties with the eradication of infection (35). Treatment with an antibiotic active against persister cells may therefore assist in preventing the formation of a chronic infection. Here, persister cell assays were performed for B. pseudomallei strains K96243 and 576. These data were analyzed using a repeated-measures (accounting for experimental runs) analysis of variance (ANOVA). There were significantly lower proportions of persister cells observed for strain 576 when incubated with finafloxacin than with ciprofloxacin at both pH 5 and pH 7 (*P* < 0.05). No persister cells were recovered at pH 5 when incubated with finafloxacin (Fig. 4). For strain K96243, there were lower proportions of persister cells at pH 7 than at pH 5 with both antibiotics (*P* < 0.001); there were no recoverable persister cells for strain K96243 when incubated with finafloxacin at pH 7.

### Pharmacokinetic studies.

The *in vitro* studies demonstrated the very promising activity of finafloxacin against B. pseudomallei, which warranted an evaluation in a murine model to determine the efficacy of the antibiotic *in vivo*. First, pharmacokinetic studies with finafloxacin were required to inform an appropriate dosing regimen for the antibiotic. Mice were dosed with a single 50-μl oral dose of 37.5 mg/kg of body weight and blood was collected at subsequent time points. Plasma was separated and analyzed for finafloxacin content by high-performance liquid chromatography (HPLC). The lowest limit of quantitation (LLOQ) for finafloxacin was 0.05 μg/ml. The oral dose selected, 37.5 mg/kg, was the highest concentration that could be delivered to the mice due to solubility constraints and using the largest volume (50 μl) that could be reproducibly delivered to a mouse orally with a pipette. The majority of finafloxacin was eliminated from the plasma by 4 h with no further detection of finafloxacin after 8 h.

The pharmacokinetic (PK) parameters were calculated from the plasma concentration time curve and compared with those from an equivalent human dose of finafloxacin of 800 mg/kg (36) (Table 2). For the fluoroquinolone class of antibiotics, the PK parameter that correlates with efficacy is the area under the concentration-time curve (AUC)/MIC (31). Therefore, to determine an equivalent human dose in the mouse, the AUC over a 24-h period was matched. The human AUC from 0 h to ∞ (AUC_0–∞_) is 29.21 h · μg/ml. Therefore, for the oral route of delivery in the mouse, this equates to 3 oral doses of 37.5 mg/kg over 24 h, giving an AUC of 33.36 h · μg/ml (Table 2).

**TABLE 2:** Comparison of PK parameters for finafloxacin in BALB/c mouse plasma and in human plasma*^a^*

PK parameter	Oral finafloxacin dose:	
	Mouse (37.5 mg/kg)	Human (800 mg/kg)
*t*_1/2_	1.89	10.47
*C*_max_ (μg/ml)	7.24	11.14
AUC_0-∞_ (h · μg/ml)	11.12	29.21

### *In vivo* efficacy study with aerosolized bacteria.

To determine the efficacy of finafloxacin against B. pseudomallei infection, BALB/c mice were dosed with a human equivalent dose of finafloxacin, ciprofloxacin, or co-trimoxazole (for comparison) following an inhalational challenge with B. pseudomallei strain K96243. Groups of 20 mice were exposed to an aerosol of B. pseudomallei over 8 runs of the apparatus. The calculated retained dose of B. pseudomallei during the runs ranged from 67 CFU to 151 CFU; therefore, the average retained dose was calculated as 128 CFU. The 50% lethal dose (LD_50_) of B. pseudomallei K96243 in BALB/c mice is 4 CFU (37); therefore, the average retained dose was equivalent to 32 LD_50_s. To enable the dosing of mice by the oral route without causing side effects, the pH of the solution should not exceed 8.0 (38). Due to ciprofloxacin requiring a diluent of a pH >8.0 to achieve solubility, a soluble oral preparation could not be prepared that would match the human AUC, and therefore an alternate formulation of ciprofloxacin was used. The dosing regimen for ciprofloxacin was determined using the dose that matched the human AUC, which equates to 30 mg/kg delivered twice daily via intraperitoneal injection (39). The co-trimoxazole dosing regimen was determined by matching the time above MIC from previously generated data (40).

**FIG 5:**
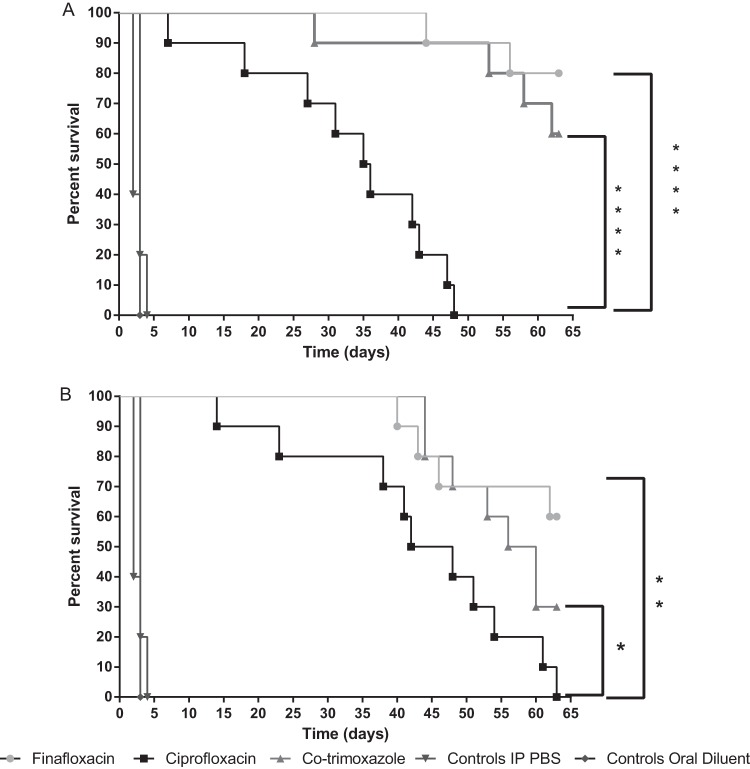
Percent survival of mice in each treatment group following a challenge with aerosolized B. pseudomallei K96243. Mice were challenged with approximately 128 CFU of B. pseudomallei K96243 by the inhalational route and treated with oral finafloxacin (37.5 mg/kg) every 8 h, intraperitoneal ciprofloxacin (30 mg/kg) every 12 h, or oral co-trimoxazole (240 mg/kg) every 12 h. Regimens were initiated at 6 h (A) or 24 h (B) postchallenge. Control animals received intraperitoneal PBS or oral diluent. Statistical analysis was performed using the Mantel-Haenszel log rank test. *, *P* = 0.043; **, *P* = 0.004; ****, *P* < 0.0001.

All control mice treated with oral diluent or phosphate-buffered saline (PBS) succumbed to infection by 96 h postchallenge (Fig. 5). When antibiotic treatment was initiated at 6 h postchallenge, the survival at day 63 was 80%, 60%, and 0% for finafloxacin, co-trimoxazole, and ciprofloxacin, respectively. All antibiotics offered greater levels of protection than oral diluent or PBS (*P* < 0.0001). Both finafloxacin and co-trimoxazole treatments afforded significantly greater survival than ciprofloxacin (*P* < 0.0001).

When antibiotic treatment was initiated later at 24 h postchallenge, the resulting survival at day 63 was 60%, 30%, and 0% for finafloxacin, co-trimoxazole, and ciprofloxacin, respectively. All antibiotics offered greater levels of protection than oral diluent or PBS (*P* < 0.0001), and both finafloxacin (*P* = 0.004) and co-trimoxazole (*P* = 0.043) afforded significantly greater survival than ciprofloxacin. There was no difference in survival when finafloxacin or co-trimoxazole was initiated at 6 or 24 h postchallenge (*P* = 0.373 or *P* = 0.283, respectively). However, survival was greater when ciprofloxacin treatment was delayed until 24 h, rather than 6 h, postchallenge (*P* = 0.044).

**FIG 6:**
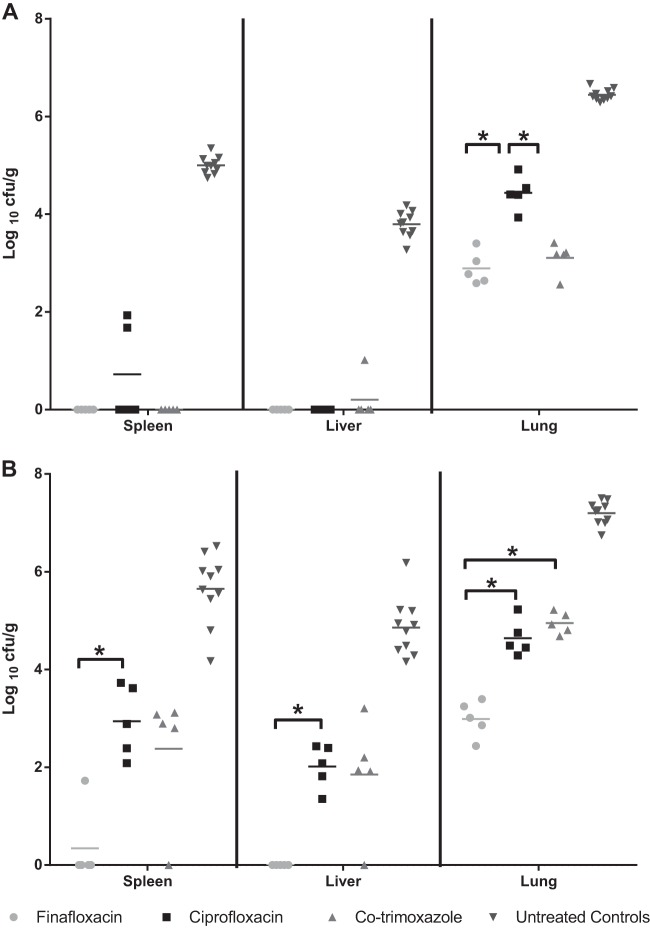
Bacterial loads in organs at 24 h following initiation of antibiotic treatment. Bacterial counts (CFU/g) in the spleens, livers, and lungs of 5 mice per group at 30 h (A) or 48 h (B) postchallenge. Each group received 24-h treatment with finafloxacin (37.5 mg/kg every 8 h orally), co-trimoxazole (240 mg/kg every 12 h orally), or ciprofloxacin (30 mg/kg every 12 h via intraperitoneal injection). Control animals received intraperitoneal PBS or oral diluent. Statistical analysis was performed using pairwise comparisons of Mood's median test. *, *P* < 0.05.

To determine the rapidity of antibiotic activity *in vivo*, five mice from each group were culled at 24 h after the onset of treatment which had been initiated at 6 or 24 h postchallenge. The spleens, livers, and lungs were harvested for the enumeration of bacterial load (Fig. 6). All antibiotic treatments afforded significantly reduced bacterial loads in the spleens, livers, and lungs in comparison to those in organs taken from control mice (*P* < 0.05), regardless of initiation at 6 or 24 h postchallenge. When finafloxacin was initiated at 6 h postchallenge, no bacteria were detected in the spleens or livers of mice. By comparison, two mice treated with ciprofloxacin and one mouse treated with co-trimoxazole had B. pseudomallei organisms detectable in the spleens and liver, respectively. Additionally, no B. pseudomallei was recovered from the remaining spleen or liver homogenates from mice treated with finafloxacin, whereas bacteria were detected in the liver homogenates of 3 mice treated with co-trimoxazole and from all mice treated with ciprofloxacin. However, overall, there was no significant difference in the bacterial loads in the spleens and livers of mice treated with finafloxacin, ciprofloxacin, or co-trimoxazole (*P* > 0.05). However, finafloxacin and co-trimoxazole afforded significantly reduced bacterial loads in the lungs in comparison to those with ciprofloxacin (*P* < 0.05).

When the antibiotic treatment was delayed until 24 h postchallenge, the mice treated with finafloxacin had significantly lower levels of bacteria in the livers and spleens in comparison to those treated with ciprofloxacin (*P* < 0.05) and lower bacterial loads in the lungs compared to those with both ciprofloxacin and co-trimoxazole (*P* < 0.05). Following the incubation of the remaining homogenates, two of the mice treated with finafloxacin appeared to have cleared the infections from all organs; however, B. pseudomallei was recovered from the other 3 animals treated with finafloxacin. B. pseudomallei was also recovered from the spleens and livers of all surviving animals treated with co-trimoxazole. There was no significant difference in the bacterial loads in the spleens, livers, or lungs of mice treated with ciprofloxacin and co-trimoxazole (*P* > 0.05).

**FIG 7:**
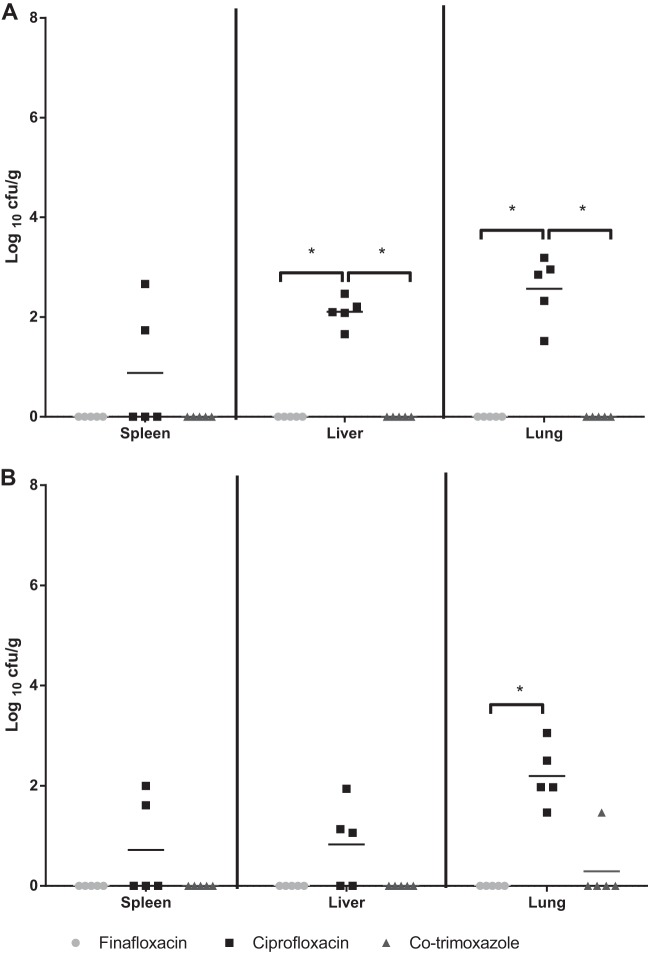
Bacterial loads in organs at the end of antibiotic treatment. Bacterial counts (CFU/g) in the spleens, livers, and lungs of 5 mice per group at 14 days posttreatment, when treatment commenced at 6 h (A) or 24 h (B) postchallenge. Each group received 14 days of treatment with finafloxacin (37.5 mg/kg every 8 h orally), co-trimoxazole (240 mg/kg every 12 h orally), or ciprofloxacin (30 mg/kg every 12 h via intraperitoneal injection). Statistical analysis was performed using pairwise comparisons of Mood's median test. *, *P* < 0.05.

At cessation of therapy, five mice from each group were culled; all mice had received 14 days of treatment initiated at 6 or 24 h postchallenge. The spleens, livers, and lungs were harvested from these animals for the enumeration of bacteria (Fig. 7). When treatment was initiated at 6 h postchallenge, there were no recoverable bacteria detected in the spleens, livers, or lungs of mice treated with either finafloxacin or co-trimoxazole. However, bacteria were recovered from the livers and lungs of all five mice and in two spleens of the animals treated with ciprofloxacin. Mice treated with finafloxacin and co-trimoxazole had significantly fewer bacteria recovered in the lungs and livers in comparison to those from mice treated with ciprofloxacin (*P* < 0.05); there was no difference between the efficacies of the three antibiotics detected in the spleens. Importantly, no B. pseudomallei was recovered from the remaining organ homogenates from mice treated with finafloxacin. By comparison, B. pseudomallei was detected in the lungs of two animals treated with co-trimoxazole and from all mice treated with ciprofloxacin.

When treatment was delayed for 24 h, there were no recoverable bacteria in the spleens, livers, or lungs of the mice treated with finafloxacin. There were no recoverable bacteria in the spleens and livers of animals treated with co-trimoxazole, and the lungs of only 1 mouse were colonized. By comparison, B. pseudomallei was recovered from all animals treated with ciprofloxacin. Overall, there were a significantly lower bacterial load in the lungs of mice treated with finafloxacin than in those treated with ciprofloxacin (*P* < 0.05), but there were no further differences in bacterial loads in the mice given different antibiotics. No B. pseudomallei was recovered from the remaining organ homogenates of mice treated with finafloxacin, and the liver and lungs of one mouse treated with co-trimoxazole were colonized. By contrast, at least one organ in each of the five animals treated with ciprofloxacin was colonized with B. pseudomallei.

**FIG 8:**
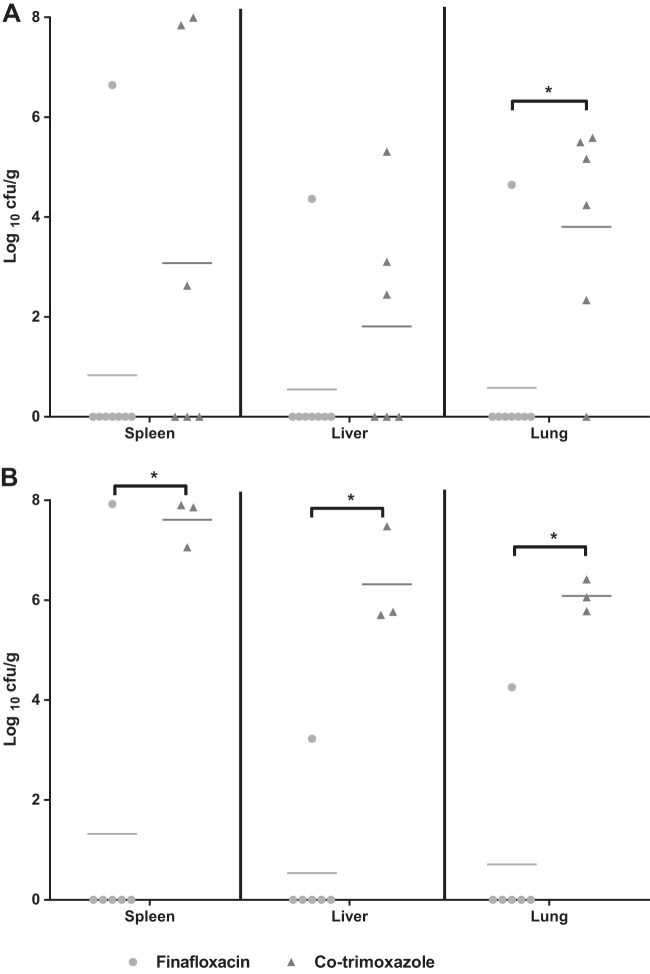
Bacterial loads in organs at 63 days postchallenge with aerosolized B. pseudomallei. Bacterial counts (CFU/g) in the spleens, livers, and lungs of mice at day 63 postchallenge when treatment commenced at 6 h (A) or 24 h (B) postchallenge. Each group received 14 days of treatment with finafloxacin (37.5 mg/kg every 8 h orally), co-trimoxazole (240 mg/kg every 12 h orally), or ciprofloxacin (30 mg/kg every 12 h via intraperitoneal injection). Statistical analysis was performed using pairwise comparisons of Mood's median test. *, *P* < 0.05.

At day 63, all ciprofloxacin-treated mice had succumbed to infection. The survivors in the finafloxacin-or co-trimoxazole-treated groups were culled and organs were harvested for the enumeration of B. pseudomallei (Fig. 8). When treatment was initiated at 6 h postchallenge, only the spleen, liver and lungs of one of the surviving eight mice treated with finafloxacin was colonized with bacteria, and one other animal had detectable B. pseudomallei in the lungs following incubation of the remaining organ homogenate. Of the surviving six mice that were treated with co-trimoxazole, at least one organ in five of these was colonized with B. pseudomallei, and the remaining organ homogenate of the sixth mouse was also shown to be colonized when it was incubated. Overall, finafloxacin afforded significantly reduced bacterial loads in the lungs compared with those with co-trimoxazole (*P* < 0.05).

When treatment was delayed for 24 h, one of the surviving six mice treated with finafloxacin was colonized with B. pseudomallei, and one other animal had B. pseudomallei recovered from the spleen homogenate. All organs tested from all surviving mice that were treated with co-trimoxazole were colonized with B. pseudomallei. When therapy was delayed for 24 h, finafloxacin was significantly more effective than co-trimoxazole in reducing bacterial loads in the spleen, liver, and lungs (*P* < 0.05).

## DISCUSSION

Melioidosis is intrinsically difficult to treat, involving an acute parenteral phase of antibiotics followed by lengthy oral eradication therapy (14). Furthermore, resistance to co-trimoxazole has been reported and it is therefore important to evaluate new antibiotics for use as postexposure prophylaxis or treatment (41).

The novel fluoroquinolone finafloxacin has potent bactericidal activity against a range of Gram-positive and Gram-negative organisms under acidic conditions (21–23, 29). Here, we have shown that finafloxacin has greatly enhanced bactericidal activity against B. pseudomallei compared with that of ciprofloxacin under acidic conditions, demonstrated by superior activity in MIC, MBC, and time-kill assays. Finafloxacin also shows increased intracellular killing compared with that of ciprofloxacin at 4× MIC in a macrophage cell infection model. Ciprofloxacin did show significant activity compared with that in untreated bacteria, possibly due to B. pseudomallei replicating in the cytoplasm where the pH is neutral (42) and ciprofloxacin is active. This could be investigated using labeled bacteria and microscopy to determine in which cellular compartment B. pseudomallei is more readily killed, supporting rational antibiotic therapy approaches. Similarly, the Bsa type III secretion system mutant, which exhibits defects in vacuole escape and therefore cannot replicate within the cytoplasm (43), could be utilized to help verify where bacteria are killed within the cell.

The conventional investigation of inhibitory and bactericidal activities of an antibiotic is carried out in medium at pH 7 (44, 45). Unfortunately, no interpretive criteria for B. pseudomallei zone diameters have been published by the CLSI, which recommends broth microdilution testing for B. pseudomallei against all antimicrobials; at an MIC of 4 μg/ml at pH 7, finafloxacin would not be recommended as therapy to treat melioidosis (14). However, at pH 5 the activity of finafloxacin against B. pseudomallei is greatly enhanced, and this may be relevant to activity in the acidic intracellular niches in which B. pseudomallei resides *in vivo*. This highlights the limitations of conventional testing methods and how the mechanism of action of each antibiotic should be considered when determining *in vitro* activity.

This study has demonstrated that finafloxacin provides a higher level of protective efficacy than ciprofloxacin in an inhalational mouse model of B. pseudomallei. When comparing finafloxacin with co-trimoxazole treatment, there was no significant difference in survival when therapy was initiated at 6 h or 24 h. However, delaying therapy with ciprofloxacin until 24 h resulted in greater survival than initiating treatment at 6 h. It is not clear why this is, and the experiment may need to be replicated to confirm the result, but one possible explanation may be that ciprofloxacin suppresses interferon gamma (IFN-γ) and interleukin-12 (IL-12) production (46). Since an immune response is generated at 10 to 24 h after inhalational infection with B. pseudomallei K96243 in BALB/c mice (47), treating with ciprofloxacin at 6 h postchallenge may suppress the initial immune response to infection, affecting the overall outcome of antibiotic treatment. Further studies are required to investigate the timing of ciprofloxacin treatment. Ultimately, all mice treated with ciprofloxacin succumbed to infection, suggesting that ciprofloxacin provides limited benefit when used as a prophylaxis against inhalational melioidosis.

At 24 h after the initiation of treatments at 6 h postchallenge, both finafloxacin and co-trimoxazole reduced the concentrations of B. pseudomallei within the lungs to levels lower than for ciprofloxacin. Finafloxacin was also better than both ciprofloxacin and co-trimoxazole at reducing the bacterial load in the lungs when therapy was delayed until 24 h postchallenge. At the end of antibiotic therapy, no bacteria were recovered from the livers and lungs of mice treated with finafloxacin or co-trimoxazole when treatments were initiated at 6 h postchallenge. Similarly, when treatment was commenced at 24 h postchallenge, B. pseudomallei was not recovered from the organs of mice treated with finafloxacin. Only the lungs of one mouse treated with co-trimoxazole were colonized with B. pseudomallei, demonstrating that both finafloxacin and co-trimoxazole are more effective than ciprofloxacin for the treatment of inhalational melioidosis in the mouse model.

When comparing the fluoroquinolones tested, finafloxacin provided better protection against B. pseudomallei than ciprofloxacin. Mice treated with ciprofloxacin started to relapse with infection at the end of the treatment regimen, whereas the mice treated with finafloxacin did not relapse at this time and continued to gain weight (data not shown); relapse in these animals was not observed until day 45 or day 40 when treatment was initiated at 6 or 24 h postchallenge, respectively. Further evidence from the bacterial burden data show that a 14-day treatment with ciprofloxacin was not able to completely kill B. pseudomallei in vivo, whereas finafloxacin appeared to be able to clear bacteria from the spleens, livers, and lungs in several animals. These data are consistent with clinical findings for ciprofloxacin where relapse or treatment failure has been observed (18). The differential activities of finafloxacin and ciprofloxacin may be related to finafloxacin having increased activity and ciprofloxacin having reduced activity at an acidic pH. Ciprofloxacin would likely have bacteriostatic activity in the acidic membrane-bound phagosomes where B. pseudomallei resides; therefore, once treatment has ceased, it is likely that B. pseudomallei is able to replicate, resulting in a relapse of infection. Under the same conditions, finafloxacin would likely be bactericidal and, as demonstrated in this study, would be able to kill bacteria and control infection (10).

For finafloxacin to control B. pseudomallei at the site of infection in the lung, it is imperative that the drug crosses the relatively impermeable alveolar membrane to penetrate the alveolar space and is taken up by alveolar macrophages from the epithelial lining fluid (ELF) and serum. Published data on lung-tissue concentrations of finafloxacin are not yet available as the agent is in early clinical development. However, distribution studies with radiolabeled finafloxacin in rats and mice indicated comparable lung-tissue concentrations for finafloxacin and moxifloxacin. These findings are nicely supported by the almost complete clearance of B. pseudomallei from the lungs of inhalant-infected mice in this study. Further studies would need to be performed to confirm this.

Although there was no significant difference in the ultimate survival of mice treated with finafloxacin and co-trimoxazole, an analysis of bacterial burden at day 63 demonstrated that the spleens, livers, and lungs from 75% of mice treated with finafloxacin from 6 h postchallenge and from 66% of mice treated with finafloxacin from 24 h postchallenge were not colonized with B. pseudomallei whereas those from all mice treated with co-trimoxazole were colonized. Furthermore, the finafloxacin-treated mice did not show any signs of infection at the end of the study. By contrast, at least one organ from all of the surviving mice that were treated with co-trimoxazole had recoverable B. pseudomallei when treatment was initiated at 6 h postchallenge; when treatment was delayed to 24 h postchallenge, the spleens, livers, and lungs of all of the mice were colonized. Therefore, although treatment with finafloxacin appears to provide no benefit compared with co-trimoxazole in terms of survival, there are apparent differences in bacterial clearance in the organs at the end of the antibiotic regimen and at day 63 of the experiment. Mice treated with co-trimoxazole also showed clinical signs of infection (data not shown), which is suggestive of a chronic infection; the majority of the mice showed splenomegaly with multiple abscesses, which is consistent with the pathology reported in human melioidosis (48).

Relapse is reported in approximately 10% of human cases of melioidosis, even after intensive antibiotic therapy (12). Despite considerable research, the mechanism by which B. pseudomallei causes latent infection is not clearly understood but is generally attributed to the many virulence factors B. pseudomallei possesses, including the type III secretion system, capsular polysaccharide, type IV pili-mediated adherence, and quorum-sensing molecules (15). B. pseudomallei is also reported to form persister cells, with some subpopulations remaining viable for at least 1 year. Although their role in the relapse of infection has not been determined, it is possible that these persister cells are involved (35). In this study, we have shown finafloxacin to be active against persister cells, another benefit that warrants further investigation of this antibiotic. However, the site of relapse for melioidosis has not been determined and may depend on the route of infection and the initial establishment of bacteria. In this study, only 3 organs were harvested; it would be useful in the future to collect other organs to determine whether they are also cleared of bacterial colonization after finafloxacin treatment. This could be particularly important for sites where antibiotic penetration is limited, such as the bone marrow where B. pseudomallei has been detected in humans or the brain, which (in mice) was shown to be colonized, particularly following an inhalational challenge (49, 50). Considering at least one organ from all of the surviving mice treated with co-trimoxazole for 14 days was colonized with B. pseudomallei and chronic infections are known to be established even after 21 days of co-trimoxazole treatment in mice (40), longer treatment regimens may be warranted than are currently recommended (17, 20, 40). Treating for longer with finafloxacin may further improve the results demonstrated in this study.

In summary, alternatives to the current therapies used to treat melioidosis are warranted. Resistance to co-trimoxazole, although rare, has also been reported in combination with adverse side events described in humans (12, 17). Finafloxacin has rapid bactericidal activity under acidic conditions against B. pseudomallei, and this aligns with significant *in vivo* protection when delivered as oral PEP in a murine inhalational model of melioidosis, offering better protection than ciprofloxacin and comparable, or better, efficacy to co-trimoxazole. Furthermore, there is evidence that 14 days of treatment with finafloxacin can prevent the establishment of a chronic infection. The unique property of finafloxacin being potent and active under acidic conditions may offer an advantage over other fluoroquinolones for treating intracellular infections. Thus, finafloxacin has the potential to provide therapeutic treatment options for melioidosis or PEP for B. pseudomallei in the event of a deliberate release or accidental exposure.

## MATERIALS AND METHODS

### Antibiotics.

Finafloxacin was supplied by MerLion Pharmaceuticals Pte Ltd. Ciprofloxacin powder was purchased from Sigma-Aldrich (UK) and an intravenous formulation of ciprofloxacin was purchased from Bayer (UK) for use in the *in vitro* and *in vivo* assays, respectively. A liquid formulation of gentamicin and an oral suspension of co-trimoxazole (Septrin) were purchased from Sigma-Aldrich (UK) and GlaxoSmithKline (UK), respectively.

### *In vitro* assays.

Working concentrations of finafloxacin or ciprofloxacin at 10 mg/ml were prepared by adding 100 mg of antibiotic to 9 ml of sterile water and 1 ml of 1 M sodium hydroxide. Bacteria grown in the equivalent concentration of sodium hydroxide used to prepare the antibiotics were included as a control. This control was included in all *in vitro* assays.

### *In vivo* study.

A 15-mg/ml solution of finafloxacin was prepared by adding 2.1 ml of 0.01 M Tris to 44 mg of finafloxacin powder (containing 37.5 mg of active ingredient). Then, 200 μl of 1 M sodium hydroxide was added to dissolve the antibiotic followed by 200 μl of 0.01 M hydrochloric acid. The pH of the resulting solution was 8; a new solution was prepared for each time point.

### Bacteria.

All bacteriological procedures were carried out in a class III microbiological safety cabinet or a class III half-suit rigid isolator within an Advisory Committee on Dangerous Pathogens (ACDP) containment level 3 laboratory. B. pseudomallei strains K96243, 576, and the mutant strain AI (deficient in the *amrA* gene, this strain was generated at Dstl, UK) were cultured in Luria broth (L broth) and incubated at 37°C with shaking at 180 rpm for 24 h.

### MICs.

MICs for finafloxacin and ciprofloxacin were determined for B. pseudomallei strains K96243 and 576 using the broth microdilution method in accordance with the Clinical and Laboratory Standards Institute (CLSI) guidelines (44). Assays were performed in 96-well microtiter plates in MHB adjusted to pH 5 or pH 7, with antibiotic concentrations in the range of 64 μg/ml to 0.03 μg/ml, and bacteria at a final concentration of approximately 5 × 10^5^ CFU/ml.

### MBCs.

MBCs for finafloxacin and ciprofloxacin were determined by plating 100-μl aliquots (derived from a total volume of 200 μl/well) of the MIC dilutions showing no visible growth onto Luria agar (L agar) plates in triplicates and incubating at 37°C for 48 h. The MBC was recorded as the lowest concentration of antibiotic that killed 99.9% of the bacteria in the original inoculum.

### Time-kill assays.

Time-kill assays were performed at 4× MICs for finafloxacin and ciprofloxacin at pH 7 (Table 1) in accordance with the CLSI guidelines (45). Antibiotic solutions of finafloxacin and ciprofloxacin were prepared in 10 ml of L broth adjusted to pH 5 or pH 7. Broths were inoculated with B. pseudomallei K96243 or 576 at a concentration of approximately 5 × 10^5^ CFU/ml; control bacteria grown in the absence of antibiotic were also included. All broths were incubated shaking at 180 rpm at 37°C. Samples were taken at 0, 2, 4, 6, and 24 h, and 10-fold serial dilutions of the samples were performed in sterile PBS, plated onto L agar, and incubated at 37°C. Plates were enumerated after incubating for 48 h. The limit of detection was approximately 5 CFU/ml. A bactericidal effect was defined as a 3-log_10_ reduction or greater in CFU/ml compared with the original inoculum; a bacteriostatic effect was defined as up to a 3-log_10_ reduction in CFU/ml compared with the original inoculum.

### Macrophage assays.

Ten milliliters of L broth was inoculated with B. pseudomallei strain AI and incubated at 37°C with shaking at 180 rpm for 24 h. The culture was adjusted to an optical density at 590 nm (OD_590_) of 0.40 and diluted 1 to 100 in Leibovitz-15 (L-15) medium (Gibco Life Technologies) supplemented with 5% fetal calf serum (FCS). A 10-fold serial dilution in PBS was performed before plating on L agar to confirm the concentration of bacteria used in the infection assay. Twenty four-well microtiter plates were seeded with 4 × 10^5^ J774 mouse macrophages (European Collection of Cell Cultures) in Dulbecco's modified Eagle's (D-MEM) medium (Gibco Life Technologies) and incubated at 37°C for 24 h. The medium was removed and replaced with 500 μl of bacteria and incubated at 37°C for 30 min. This was removed and replaced with 500 μl of 10 mg/liter gentamicin in L-15 medium containing 5% FCS to kill the extracellular bacteria. The gentamicin was removed after 30 min (time zero) and was replaced with 1 ml of L-15 medium or 1 ml of antibiotic (at 1× or 4× the MIC) in L-15 media. At time zero and 24 h postinfection, the cells were lysed with distilled water and a 10-fold serial dilution was performed in PBS and plated on L agar. Bacteria were enumerated following incubation at 37°C for 48 h.

### Persister cell assays.

Ten milliliters of L broth adjusted to pH 5 or pH 7 was inoculated with B. pseudomallei strain K96243 or 576 and incubated at 37°C with shaking at 180 rpm for 24 h. The cultures were then adjusted to an OD_590_ of 0.40 to obtain a culture of approximately 10^8^ CFU/ml. Bacterial enumeration was performed to confirm the bacterial starting concentration (input). Antibiotic solutions were prepared at 100× the MIC; finafloxacin and ciprofloxacin were prepared in 500 μl aliquots in a 24-well microtiter plate containing L broth adjusted to pH 5 or pH 7. Wells were inoculated with 500 μl of adjusted B. pseudomallei K96243 or 576; control bacteria grown in the absence of antibiotic were also included. The microtiter plates were incubated at 37°C for 24 h, and then transferred to tubes and centrifuged at 5,000 rpm for 8 min. The supernatants were removed and a 10-fold serial dilution was performed in PBS and plated on L agar. The pellet was resuspended in 1 ml of L broth; a 10-fold serial dilution was performed in PBS and plated on L agar plates to determine the bacterial concentration (output). Bacteria were enumerated following incubation at 37°C for 48 h. The persister cell percentages were determined by dividing the input CFU/ml by the output CFU/ml and multiplying by 100.

### Animals.

Animal studies were carried out in accordance with the UK Animals (Scientific Procedures) Act of 1986 and the Codes of Practice for the Housing and Care of Animals Used in Scientific Procedures of 1989. Female BALB/c mice (Charles River Laboratories, UK) aged 8 to 10 weeks were randomized into cages of 5 within an ACDP level 2 laboratory for the pharmacokinetic studies or a class III half-suit rigid isolator in an ACDP level 3 laboratory for the efficacy studies. Mice had free access to water and rodent diet (Harlan Teklad, UK) and underwent a 7-day acclimatization period before any procedures were performed.

### Pharmacokinetic studies.

Groups of 5 mice were administered single 50-μl oral doses of 37.5 mg/kg of finafloxacin via a pipette. Blood was collected via cardiac puncture under terminal anesthesia into lithium heparin tubes at 0, 0.5, 1, 1.5, 2, 2.5, 3, 4, 5, 6, and 8 h after dosing. The plasma was separated by centrifugation at 5,000 rpm. Plasma samples were analyzed by HPLC by Swiss BioQuant AG (Switzerland). The mean finafloxacin concentration-time profile was generated from the plasma concentrations, and PK analysis of these profiles was completed using WinNonlin Phoenix v 6.1 (Pharsight Corp., St. Louis, MO) to calculate the maximum drug concentration in serum (*C*_max_), AUC, and the terminal half-life (*t*_1/2_). The results were used to determine the dosing regimen for the efficacy study.

### *In vivo* efficacy study with aerosolized bacteria.

B. pseudomallei strain K96243 was prepared by adding 10 μl of a frozen bacterial stock to 75 ml of L broth and incubating at 37°C with shaking at 180 rpm for 24 h. The culture was adjusted to an OD_590_ of 0.40 to obtain approximately 10^8^ CFU/ml. The culture was diluted 1:60 for use in the efficacy study. To administer the bacteria, mice were restrained within a nose-only exposure tube and placed within an animal exposure chamber, which was connected to a Collison three-jet nebulizer and spray tube in a Henderson apparatus. Ten milliliters of bacteria was placed into the nebulizer and mice were exposed for 10 min to a dynamic aerosol conditioned in the Henderson apparatus (51). The aerosol stream was maintained at 50 to 55% relative humidity and 22°C; the particle size generated was within the range of 1 to 3 μm. The concentration of B. pseudomallei in the aerosol was determined by recovering samples from the exposure chamber using an all-glass impinger operating at 12 liter/min, containing 10 ml of sterile PBS. Impinger samples were plated on L agar for bacterial enumeration, and the calculated retained dose of bacteria that mice received in each run was calculated by applying the Guyton formula (52). It was assumed that each mouse retained 40% of the organisms that were inhaled (53).

Therapy was initiated at 6 or 24 h postchallenge. Groups of 20 mice were administered finafloxacin (37.5 mg/kg) in a 50-μl oral dose via pipette every 8 h, ciprofloxacin (30 mg/kg) in a 300-μl intraperitoneal dose every 12 h, or co-trimoxazole (240 mg/kg) in a 50-μl oral dose via pipette every 12 h. Control groups of infected mice were administered 50 μl diluent (consisting of Tris, sodium hydroxide, and hydrochloric acid, adjusted to pH 8) orally via pipette every 8 h, or 300 μl of sterile PBS via intraperitoneal injection every 12 h. All treatment regimens were continued for 14 days.

Mice were weighed daily and observed twice daily for clinical signs of disease for 63 days, the time point at which the experiment was terminated. Additionally, at 24 h postchallenge and at the cessation of antibiotic therapy (day 15), 5 mice from each group were culled. Postmortems were performed on all mice, and the spleens, livers, and lungs were harvested, weighed, and homogenized in 1 ml of sterile PBS. A 10-fold serial dilution was performed and 100 μl aliquots were plated on L agar in duplicates. The agar plates were incubated for 48 h at 37°C, and bacteria were enumerated to determine the bacterial loads in the organs. The remaining homogenates were placed into 10 ml of L broth and incubated at 37°C for 7 days. For organs that were clear by plate count, a 10-μl loop of the relevant incubated homogenate was streaked on L agar plates and incubated at 37°C for a further 48 h, after which the visual presence of B. pseudomallei colonies was determined.

### Statistical analysis.

All graphs were generated using GraphPad Prism v 6.02. Statistical analysis was performed using Prism with the exception of the *in vivo* bacterial colonization data which were analyzed using IBM SPSS v 21.0. Using a combination of QQ plots and Levene's and Bartlett tests for unequal variance, all bacterial count data and persister cell proportion data were found to be exponential in nature and were therefore transformed to the logarithm of 10 for further statistical analysis (data not shown). The cell culture data were analyzed by 2-way ANOVA with experimental run used as a repeated measure, and fold changes in bacterial numbers were graphed. Tukey's multiple-comparison test was used for individual comparisons. The *in vivo* survival rates were analyzed using the Mantel-Haenszel log rank test. Bacterial burdens within organs were analyzed using Mood's median test with pairwise analysis. A *P* value of < 0.05 was considered statistically significant.
